# Supplementary material for: Detection of genomic G-quadruplexes in living cells using a small artificial protein
Source: Nucleic Acids Res. 2020 Oct 12;48(20):11706–20. doi: 10.1093/nar/gkaa841 (PMC7672459; doi:10.1093/nar/gkaa841)
Supplement: gkaa841_Supplemental_File [file gkaa841_supplemental_file.pdf]

Supporting Information for

Detect Genomic G-Quadruplexes in Living Animal Cells with a Tiny Artificial Protein

Ke-wei Zheng[1,2,†], Jia-yu Zhang[1,3,†*], Yi-de He[1,4,†], Jia-yuan Gong[1], Cui-jiao Wen[1], Juan-nan Chen[2], Yu-hua Hao[1], Yong Zhao[4], Zheng Tan[1,5,*]

[1]State Key Laboratory of Membrane Biology, Institute of Zoology, Chinese Academy of Sciences, Beijing 100101, P. R. China

[2]School of Pharmaceutical Sciences (Shenzhen), Sun Yat-Sen University, Guangzhou 510275, P.R. China

[3]CAS Key Laboratory for Biomedical Effects of Nanomaterials and Nanosafety, Multidisciplinary Research Division, Institute of High Energy Physics, Chinese Academy of Sciences, Beijing 100049, P. R. China

[4]School of Life Sciences, Sun Yat-Sen University, Guangzhou 510006, P.R. China

[5]Center for Healthy Aging, Changzhi Medical College, Changzhi 046000, Shanxi, P. R. China

†Contributed equally to the work

* Address correspondence to

Jia-yu Zhang, Email: zhangjy86@ihep.ac.cn or

Zheng Tan, z.tan@ioz.ac.cn

Keywords: DNA structure, G-quadruplex, Nucleic acids, Transcription

Contents

Figure S1 .... S3
Figure S2 .... S4
Figure S3 .... S5
Figure S4 .... S6
Figure S5 .... S7
Figure S6 .... S8
Figure S7 .... S9
Figure S8 .... S10
Figure S9 .... S11
Figure S10 .... S12
Figure S11 .... S13
Figure S12 .... S14
Table S1. Sequences of DNAs used in binding and CD analysis. S15
References for Table S1 .... S16
Table S2. Sequences of PQS motifs used in plasmid pull-down. S17
Table S3. Sequences of dsDNA used in CD and EMSA analysis. S18
Table S4. G4P peaks detected in human A549 cells (rep1). S19
Table S5. ChIP-qPCR primers. S20

Figure S1

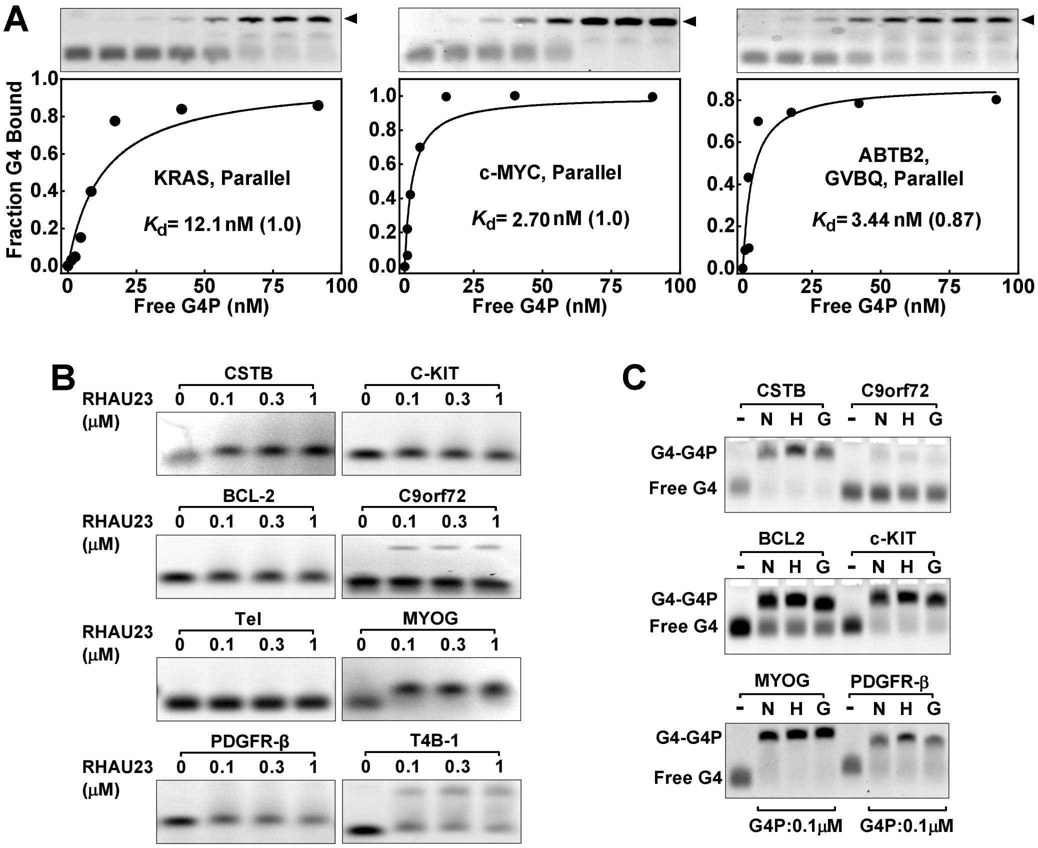

Figure S1. Additional characterization of RHAU23 and G4P by the electrophoretic mobility shift assay (EMSA). (A) Dissociation constant K_d of G4P to G4s. (B) RHAU23 bound poorly to G4s. (C) G4P binding to G4s with or without tags. G4P with tags was able to bind G4 as the G4P core. -: No G4P; N: G4P with an NLS-tag at the N terminal and a 3xFLAG tag at the C terminal; H: G4P with a HIS-tag at the N terminal and a 3xFLAG tag at the C terminal; G: G4P core without a tag.

Figure S2

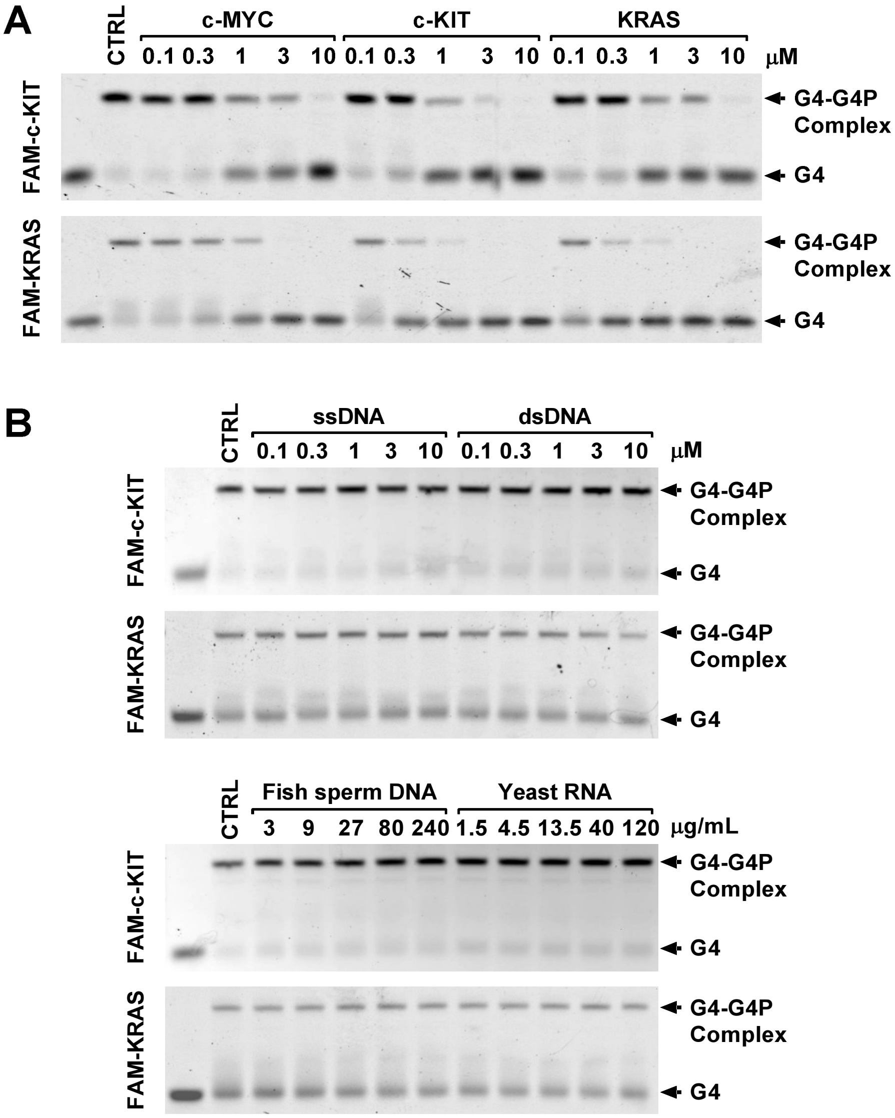

Figure S2. Specific binding of G4P to G4s assayed by binding competition. (A) Loss of G4P binding to FAM-labeled c-KIT and KRAS G4 in the presence of competitors c-MYC, c-KIT, and KRAS G4 DNA. (B) G4P binding to FAM-labeled c-KIT and KRAS G4 was not affected by non-G4 competitors. G4P was incubated with FAM-labeled c-KIT or KRAS G4 at 4 °C for 30 min, and then incubated with the indicated competitor at 37 °C for 30 min before being assayed by EMSA.

Figure S3

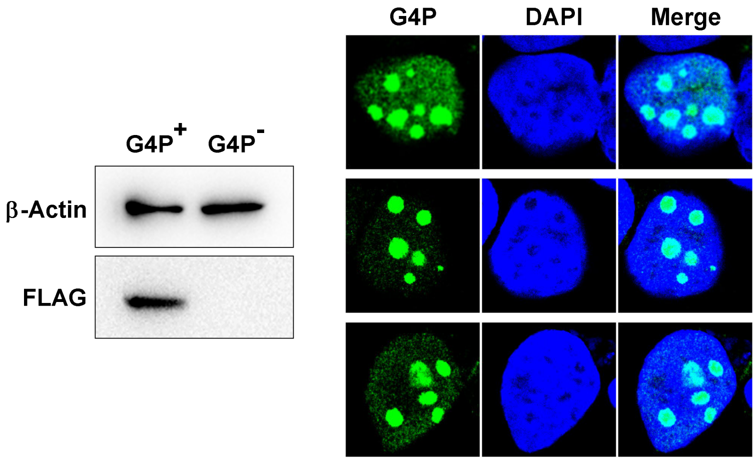

Figure S3. Expression of G4P detected by Western blot (Left) and immunofluorescent confocal microscopy (right) in 293T cells using an antibody to the FLAG of the G4P. G4P was introduced into 293T cells by site-specific gene knock-in.

Figure S4

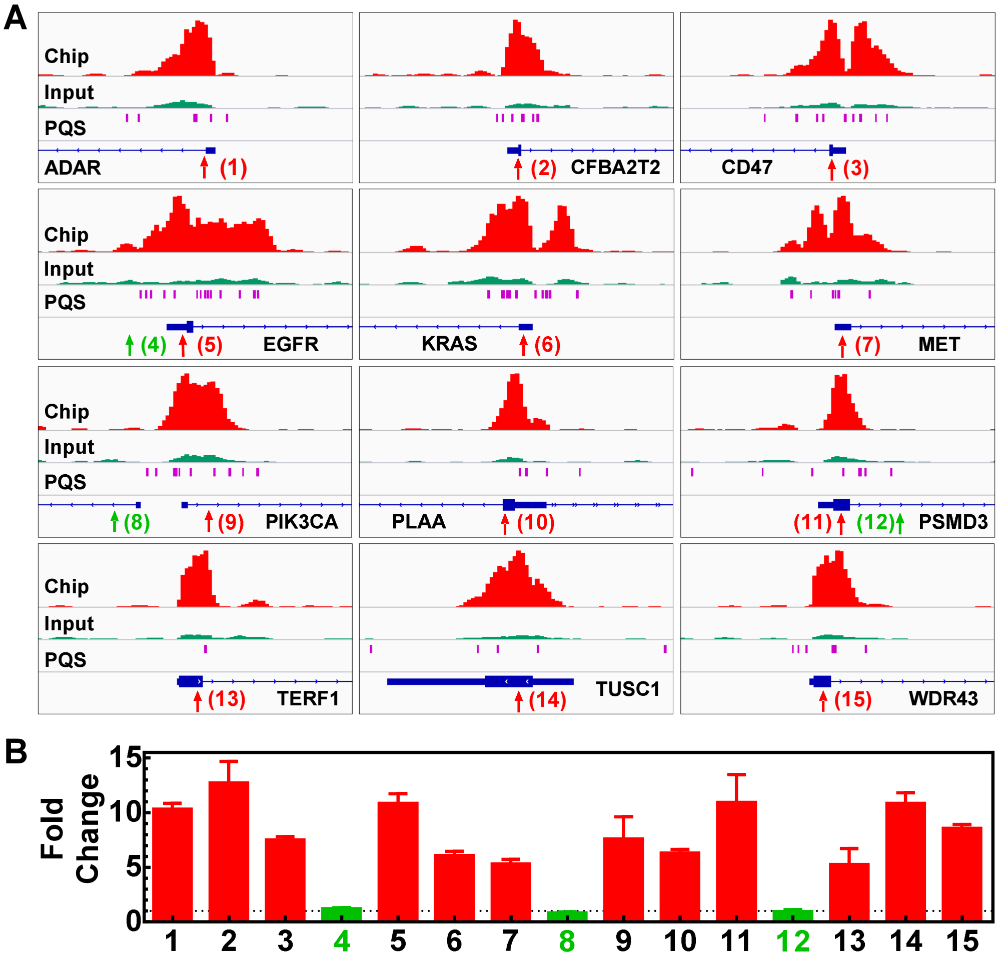

Figure S4. Verification of G4P enrichment in genomes of A549 cells by ChIP-qPCR. (A) qPCR regions are indicated by numbered arrowheads. (B) Enrichment of G4P at the indicated qPCR regions in panel A expressed as means of duplicate with range.

Figure S5

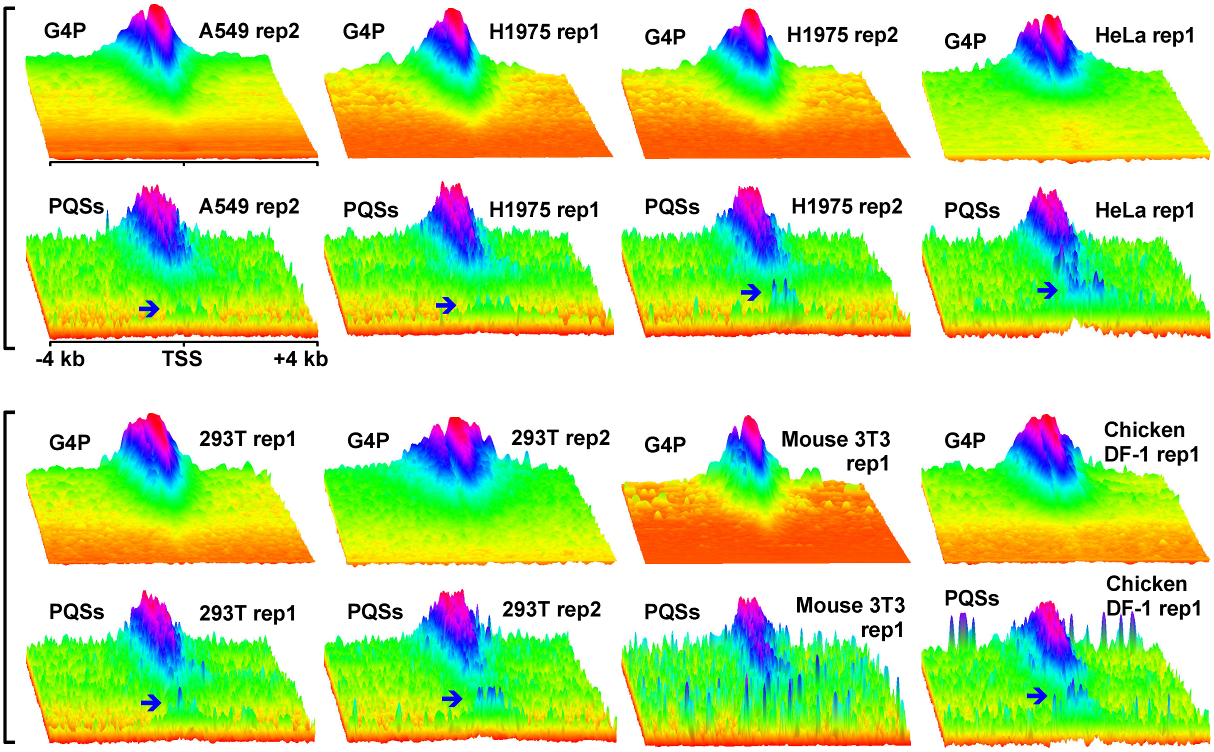

Figure S5. Overview of G4 formation and PQS distributions in A549, 293T, HeLa-S3, NCI-H1975, mouse 3T3, and chicken DF-1 cells in the TSS±4kb regions of RefSeq genes. Figure 5A and A549 rep2, 293T rep1, and rep2 were biological replicates (independent starting from cell culture); H1975 rep1 and rep2 were sequencing replicates. Data were analyzed as in Figure 5, A and B with each pair of heatmaps produced using an independent TSS region file sorted on the G4P reads. The blue arrowhead shows a small fraction of the PQS motifs with a low probability of G4 formation.

Figure S6

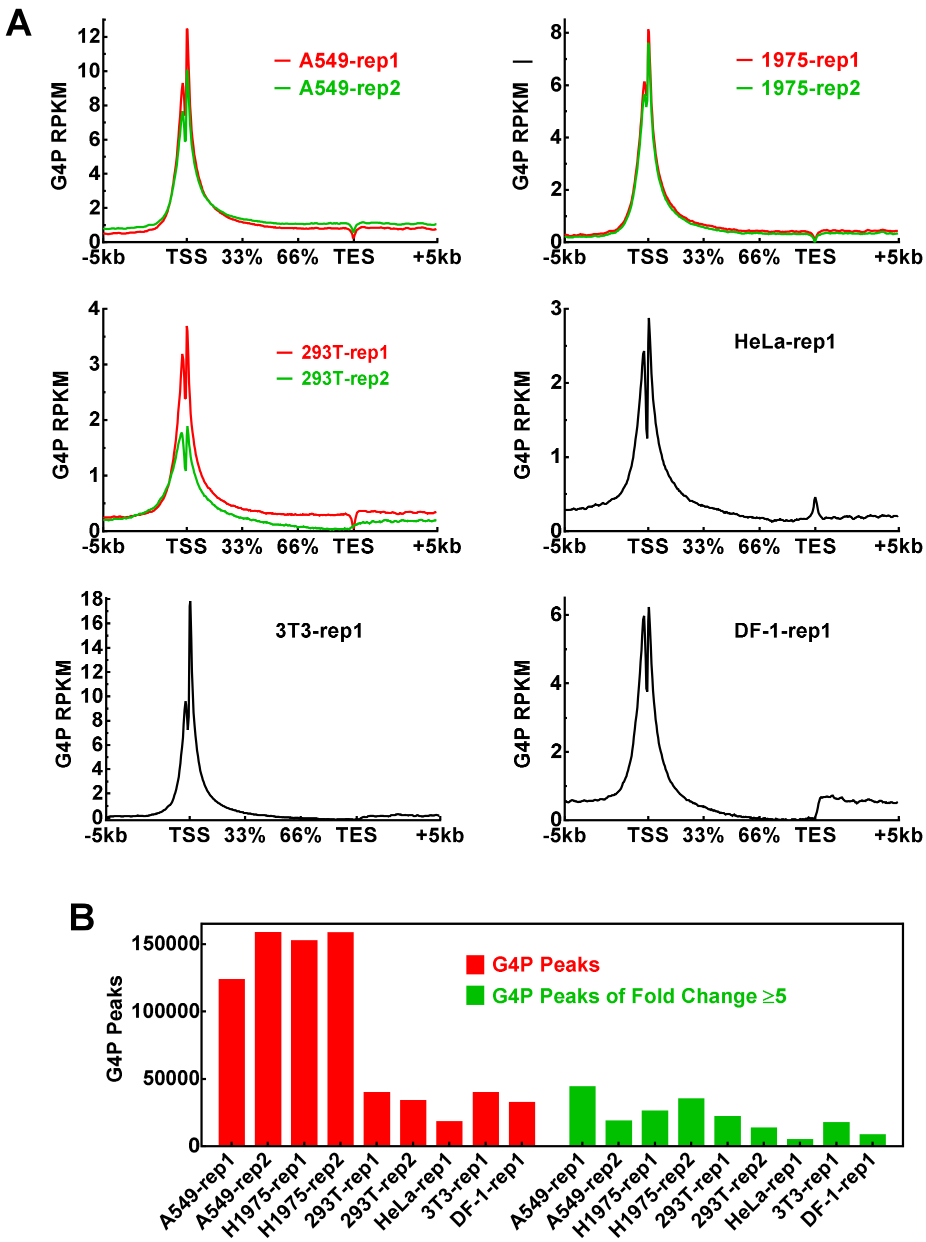

Figure S6. (A) G4P reads distribution across RefSeq genes in A549, 293T, HeLa-S3, NCI-H1975, mouse 3T3, and chicken DF-1 cells. (B) Number of G4P peaks detected in the corresponding cells. The greater variation between the different cell lines than that of the same cell line demonstrated a cell line-specific feature.

Figure S7

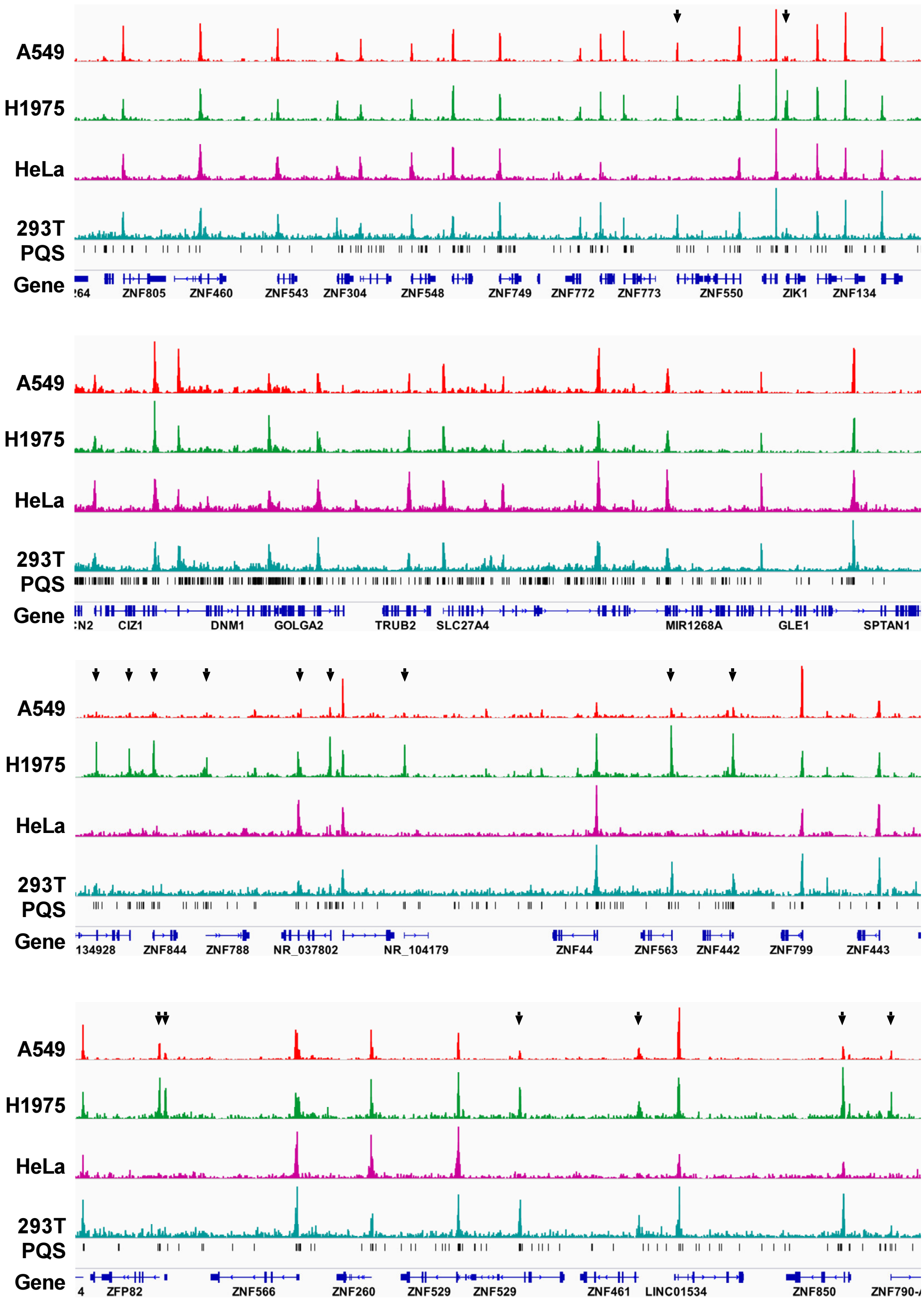

Figure S7. Examples of G4 formation in human cells depicted by the G4P peaks. Black arrowheads indicate loci where G4 formed in some cells but not in others.

Figure S8

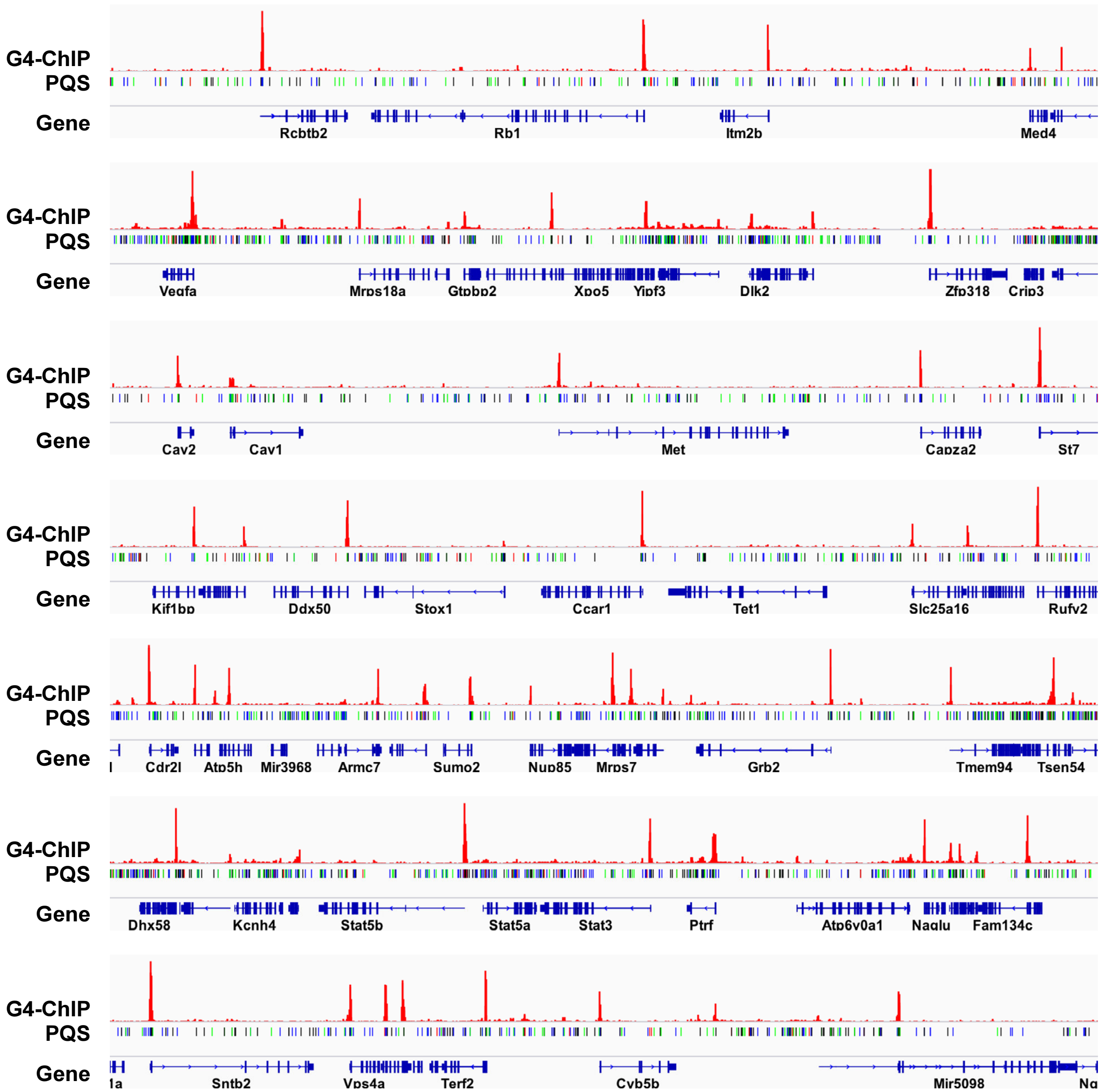

Figure S8. Examples of G4 formation in mouse 3T3 cells depicted by the G4P peaks.

Figure S9

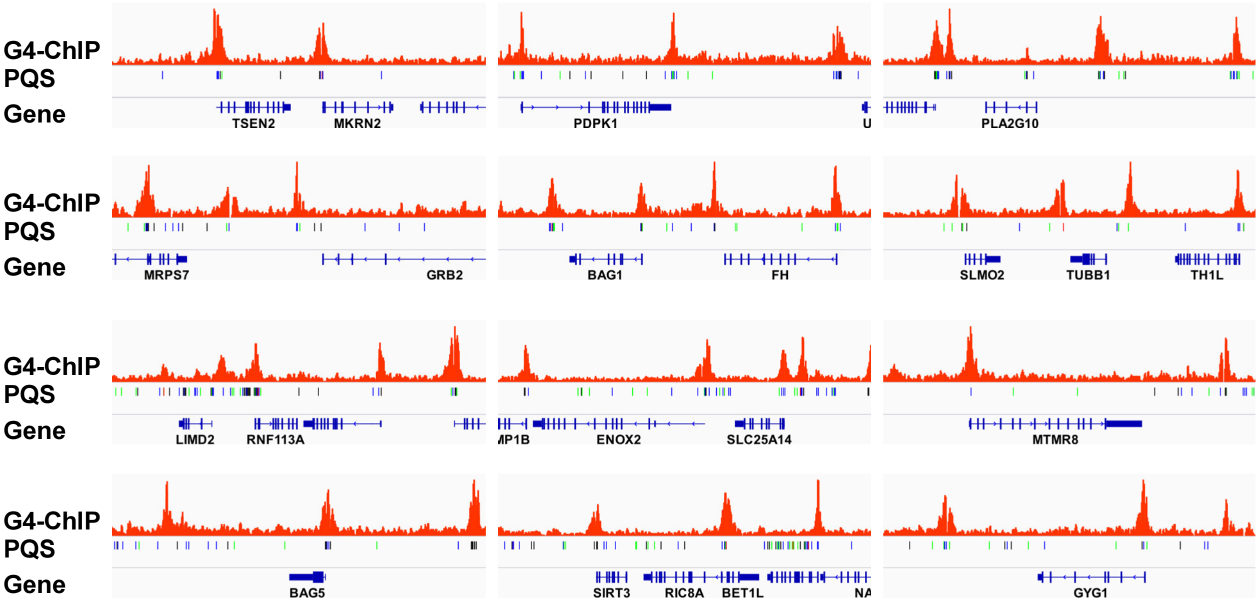

Figure S9. Examples of G4 formation in chicken DF-1 cells depicted by the G4P peaks.

Figure S10

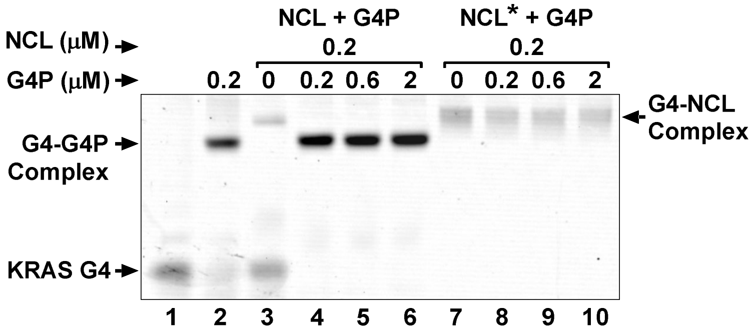

Figure S10. Fixed G4-nucleolin complexes prevent further binding by G4P. G4P was incubated at the indicated concentration with KRAS G4 (20 nM) alone (lane 2) or after the G4 was incubated for 30 min with 0.2 µM nucleolin (NCL, lanes 3-10). NCL* indicates the incubation with NCL was followed by crosslinking with 1% formaldehyde for 10 min, then terminated with 0.125 M glycine before the incubation with G4P.

Figure S11

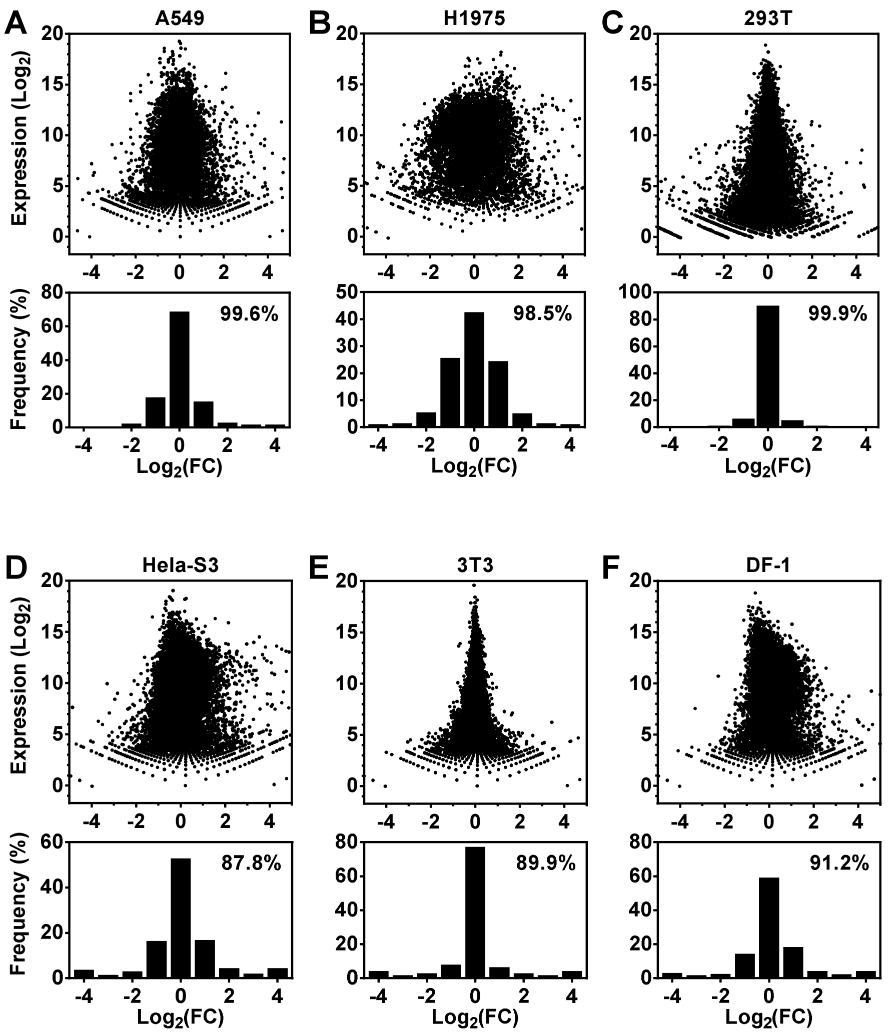

Figure S11. RNA-Seq results showing changes in gene expression in cells after the introduction of G4P. All cells, except the 293T, were transfected with a G4P expressing plasmid. G4P was introduced into 293T cells by site-specific gene knock-in. Numbers inside the panels indicate the percentage of genes whose change in RNA level is less than or equal to 2^2 (4 folds).

Figure S12

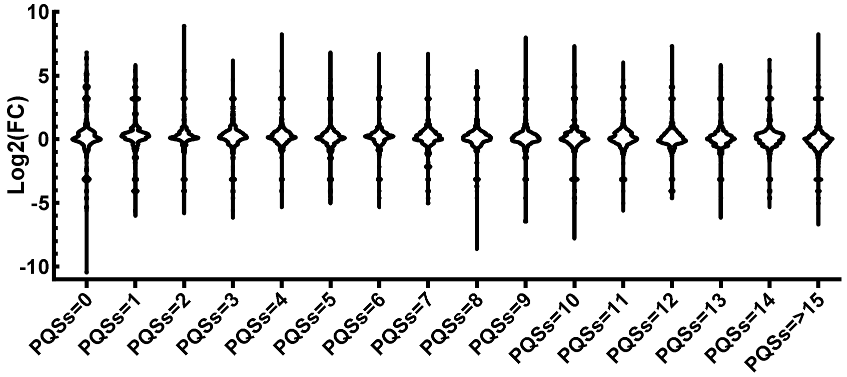

Figure S12. Variation in gene expression in cells after the introduction of G4P groupped according to the number of PQS motifs within the TSS±2kb regions. Expression data were from **Figure S11**A.

Table S1. Sequences of DNAs used in binding and CD analysis.

Name	Sequence (5' to 3')	Structure Type	Topology	Reference
CSTB	GGGGCGGGGCGCGGGGCGGGG	G4	Parallel	This work
c-KIT	TTCGGGCGGGCGCGAGGGAGGG TTTT	G4	Parallel	(1)
C9orf72	GGGGCCGGGGCCGGGGCCGGG GCC	G4	Antiparallel	(2)
BCL2	GGGCGCGGGAGGAAGGGGGCG GG	G4	Mixed parallel and antiparallel	(3)
Tel	GG(TTAGGG)4TTAG	G4	Mixed parallel and antiparallel	(4)
MYOG	AGGGTGGGCTGGGAGGT	G4, GVBQ	Parallel	(5)
PDGFR-β	AAGGGAGGGCGGCGGGGCAGG GA	G4, Broken	Parallel	(6)
T4B-1	TATTGTTTTGGTGGGTGGGTGG GTTAT	G4, Bulge	Parallel	(7)
c-MYC	AGGGTGGGGAGGGTGGGGA	G4	Parallel	(8)
KRAS	AGGGCGGTGTGGGAAGAGGGA AGAGGGGGAGG	G4	Parallel	(9)
ABTB2	TGGGCGGAGGGAAGTGGGA	G4, GVBQ	Parallel	(5)
Tel-C-rich	CCCTAACCCTAACCCTAACCCT	i-motif	NA	(10)
BCL2-C-rich	CCCGCCCCCTTCCTCCCGCGCCC	i-motif	NA	(11)
ssDNA1	CCAGCCTGCGGCGAGTGAAGAA GAGAAGAAGAGTAGA	ssDNA	NA	
ssDNA2	TCCATGGTGGCTTATGGATGT	ssDNA	NA	
DNA Hairpin1	TCAGCTGCGCAGTACAGATCTG TACTGCGCAGCTGA	DNA duplex	NA	
DNA Hairpin2	TCAGCTGCGCGCAGCGATTGCG TTTTCGCTGCGCGCAGCTGA	DNA duplex	NA	

NA, not applicable.

References for Table S1

1. Fernando, H., Reszka, A.P., Huppert, J., Ladame, S., Rankin, S., Venkitaraman, A.R., Neidle, S. and Balasubramanian, S. (2006) A conserved quadruplex motif located in a transcription activation site of the human c-kit oncogene. *Biochemistry*, **45**, 7854-7860.
2. Zhou, B., Liu, C., Geng, Y. and Zhu, G. (2015) Topology of a G-quadruplex DNA formed by C9orf72 hexanucleotide repeats associated with ALS and FTD. *Sci Rep*, **5**, 16673.
3. Dai, J., Dexheimer, T.S., Chen, D., Carver, M., Ambrus, A., Jones, R.A. and Yang, D. (2006) An intramolecular G-quadruplex structure with mixed parallel/antiparallel G-strands formed in the human BCL-2 promoter region in solution. *J. Am. Chem. Soc.*, **128**, 1096-1098.
4. Biffi, G., Tannahill, D., McCafferty, J. and Balasubramanian, S. (2013) Quantitative visualization of DNA G-quadruplex structures in human cells. *Nat. Chem.*, **5**, 182-186.
5. Li, X.M., Zheng, K.W., Zhang, J.Y., Liu, H.H., He, Y.D., Yuan, B.F., Hao, Y.H. and Tan, Z. (2015) Guanine-vacancy-bearing G-quadruplexes responsive to guanine derivatives. *Proc. Natl. Acad. Sci. USA*, **112**, 14581-14586.
6. Chen, Y., Agrawal, P., Brown, R.V., Hatzakis, E., Hurley, L. and Yang, D. (2012) The major G-quadruplex formed in the human platelet-derived growth factor receptor beta promoter adopts a novel broken-strand structure in K+ solution. *J. Am. Chem. Soc.*, **134**, 13220-13223.
7. Mukundan, V.T. and Phan, A.T. (2013) Bulges in G-quadruplexes: broadening the definition of G-quadruplex-forming sequences. *J. Am. Chem. Soc.*, **135**, 5017-5028.
8. Stump, S., Mou, T.C., Sprang, S.R., Natale, N.R. and Beall, H.D. (2018) Crystal structure of the major quadruplex formed in the promoter region of the human c-MYC oncogene. *PLoS ONE*, **13**, e0205584.
9. Cogoi, S. and Xodo, L.E. (2006) G-quadruplex formation within the promoter of the KRAS proto-oncogene and its effect on transcription. *Nucleic Acids Res.*, **34**, 2536-2549.
10. Zhao, Y., Zeng, Z.X., Kan, Z.Y., Hao, Y.H. and Tan, Z. (2005) The folding and unfolding kinetics of the i-motif structure formed by the C-rich strand of human telomere DNA. *Chembiochem*, **6**, 1957-1960.
11. Kendrick, S., Kang, H.J., Alam, M.P., Madathil, M.M., Agrawal, P., Gokhale, V., Yang, D., Hecht, S.M. and Hurley, L.H. (2014) The dynamic character of the BCL2 promoter i-motif provides a mechanism for modulation of gene expression by compounds that bind selectively to the alternative DNA hairpin structure. *J. Am. Chem. Soc.*, **136**, 4161-4171.

Table S2. Sequences of PQS motifs used in plasmid pull-down.

Motif	Sequence (5’ to 3’)
Mutant	CGTGGCGAGCGTGGGG
CSTB	GGGGCGGGGCGCGGGGCGGGG
c-MYC	GGGTGGGGAGGGTGGGG

Table S3. Sequences of dsDNA used in CD and EMSA analysis.

Name	Sequence (5' to 3')
dsCSTB G/T	CCTGAAGCAGACAGCTAGTGAATTCGGGGCGGGGCGCGGGGCGGGG TACTTGCGTATAACTGTTCCATAGT<br>ACTATGGAACAGTTATACGCAAGTATTTTTTTTTTTTTTTTTTTTTGAA TTCACTAGCTGTCTGCTTCAGG
dsCSTB	CCTGAAGCAGACAGCTAGTGAATTCGGGGCGGGGCGCGGGGCGGGG TACTTGCGTATAACTGTTCCATAGT<br>ACTATGGAACAGTTATACGCAAGTACCCCGCCCCGCGCCCCGCCCCG AATTCACTAGCTGTCTGCTTCAGG

Table S4. G4P peaks detected in human A549 cells (rep1).

This table holds more than 1000 pages for the peaks of ≥5 fold changes, so it is provided in an embedded file: Table-S4-A549-rep1-G4P-narrowPeak-FCeg5-with-PQS.xlsb.

chr	start	end	Peak	UCSC Score	strand	Fold-change	-log10pvalue	-log10qvalue	Relative summit position to peak start	4G count	4G motif	4GL15 count	4GL15 motif	Bulge count	Bulge motif	GVBQ count	GVBQ motif	All GQ count	Host Gene
chr1	805366	805556	A549-chi	335	.	10.08609	36.29663	33.5443	110	1	[CCCC]ga	0	.	1	[CCCCC]a	0	.	2	NR_027055.1/FAM41C
chr1	839153	840048	A549-chi	411	.	10.43142	43.95774	41.11716	595	3	[CCCC]tg	1	[CCCC]gg	1	g{ccgcc}	1	[CC]g[CC	6	.
chr1	840219	840858	A549-chi	236	.	8.54965	26.30185	23.68141	128	3	[GGGG]ag	0	.	0	.	1	[CCCC]g[	4	.
chr1	845438	846166	A549-chi	148	.	5.41169	17.25251	14.80033	578	3	[GGG]aa[	2	[CCC]ggg	1	[CCC]aga	2	[GG]cgcg	8	.
chr1	848471	848626	A549-chi	109	.	5.04454	13.31366	10.97215	55	0	.	1	[GGG]tgc	1	a{ggag}a	1	[GGG]ca[	3	.
chr1	854685	855197	A549-chi	195	.	6.69307	22.14046	19.58708	152	0	.	0	.	2	g{ccac}g	1	[CC]acgg	3	NR_026874.2/LOC100
chr1	855718	856520	A549-chi	138	.	5.35148	16.32461	13.89391	226	0	.	0	.	1	[CCCC]at	0	.	1	NR_026874.2/LOC100
chr1	856665	857309	A549-chi	249	.	7.28408	27.59432	24.95531	127	0	.	0	.	1	[GGGG]ct	0	.	1	NR_026874.2/LOC100
chr1	858183	861569	A549-chi	945	.	11.03173	97.96663	94.54871	1868	7	[GGG]cac	7	[CCC]acg	12	[GGGG]ca	10	[GG]aa[G	36	NM_152486.2/SAMD11
chr1	866089	866901	A549-chi	208	.	7.35351	23.40993	20.83487	318	1	[CCCC]t[	1	[CCCCC]t	1	t{cacc}t	1	[CC]t[CC	4	NM_152486.2/SAMD11

Table S5. ChIP-qPCR primers.

Gene	Sequences (5'-3')
ADAR	TGTCCTTCTCGGCTACACCTG
	CACGCTTCCTCTAACATCAACG
CBFA2T2	GCTCGGCGATGGTAGGCGT
	CCCGCATTCACGCCCCAC
CD47	TCACCGCAGCACGCCGAG
	CGGAGATGTGGCCCCTGGTA
EGFR	GAGGTGGGGACCCGAATAAA
	TGGCCGAGCCTTAGAGCC
	CGCCAACGCCACAACCA
	CGGAGGGTCGCATCGCT
KRAS	CCCGCCATTTCGGACTG
	GGAGCCGCTGAGCCTCTG
MET	GATGCGGGGCGACAGCT
	AGCGGCGCAAGGACCAC
PIK3CA	TCCGCCTTCGGGATGGTAT
	GCGTTGCTGTGCGTTCTTC
	CTTCCTTTGCTTCTACTCCCAGTT
	GCGCACTTCCTCAACCTCC
PLAA	CGGTCTCGGGACACGGACAC
	GGACGTACGGGGCCTGGTG
PSMD3	CCCCAGGATGTGGAGATGAA
	CCGTCTTGCCGTCTGCC
	CTCAACCTTTGGCCTAAACTCC
	TTGGAGGAACAAGAGGACTACAGAC
TERF1	CTCTTTGCCGAGCTTTCCG
	CACCCTCTGCGCTGTTGC
TUSC1	TCGTCCCGCGCACGGATG
	CCCGACAGCAGCTGGAGGAGC
WDR43	GTATGGGAGACGGCCAACAA
	AGGCCAGACAGGTGCAGGTA
